# Retraction: Interleukin-6 Synthesis in Human Chondrocytes Is Regulated via the Antagonistic Actions of Prostaglandin (PG)E_2_ and 15-deoxy-Δ^12,14^-PGJ_2_

**DOI:** 10.1371/journal.pone.0306507

**Published:** 2024-07-30

**Authors:** The PLOS ONE Editors

After this article [1] was published, concerns were raised regarding Figures 1, 3, 4, 7 and 8.

Specifically, when the aspect ratio and color levels are adjusted:

- The following lanes appear similar to each other:
  - In Figure 1A of [1], lanes 2–5 of the β-actin panel.
  - In Figure 1B of [1], lanes 3–6 of the β-actin panel.
  - In Figure 3C of [1], the β-actin panel.
  - In Figure 4B of [2], lanes 3–6 of the β-actin panel.
- The following lanes appear similar to each other:
  - In Figure 1B of [1], lane 1 of the β-actin panel.
  - In Figure 4B of [2], lane 1 of the β-actin panel.
- The following lanes appear similar to each other:
  - In Figure 1A of [1], lane 1 of the β-actin panel.
  - In Figure 1B of [1], lane 2 of the β-actin panel.
  - In Figure 4B of [2], lane 2 of the β-actin panel.
- The following lanes appear similar to each other:
  - In Figure 1A of [1], lane 6 of the β-actin panel.
  - In Figure 4B of [2], lane 7 of the β-actin panel.
- The p-ERK1/2 panel in Figure 4A appears similar to lanes 1–4 of the p-ERK1/2 panel in Figure 4B in [1].
- The p-CREB (ser133) panel in Figure 4A of [1] appears similar to the IL-6 panel in Figure 1A of [2].
- In Figure 7 of [1], the following appear similar:
  - The middle band in lane 6 of Figure 7B and the middle band in lane 3 of Figure 7D.
  - The upper bands in lane 1 of Figures 7B and 7D.
  - The top edges of the bottom bands of lanes 2, 3, 5 and 6 of Figure 7B and top edges of the bottom bands of lanes 2, 3, 5 and 6 of Figure 7D, respectively.
  - The upper band in lane 1 in Figure 7E and the upper band in lane 1 of Figure 7F.
  - The upper band in lane 3 in Figure 7E and the middle band in lane 4 of Figure 7F, when flipped horizontally.
- In Figure 8D of [1] the IP and Input panels appear to have similar background patterns, when exposure levels are altered.

The corresponding author indicated they are unable to recover and provide the requested underlying data. In light of the unresolved concerns affecting multiple figures, which cannot be resolved in the absence of the data underlying the published figures, the *PLOS ONE* Editors retract this article.

The Figure 1A, 1B, and 3C Beta-actin panels, and the Figure 4A p-CREB (Ser133) panel were previously published in [2] and may not have been offered under a CC-BY 4.0 license. Please refer to [2] for the correct copyright for these panels.

KK agrees with the retraction decision and apologizes for the issues with the published article. PW and FZ either did not respond directly or could not be reached.
